# Assessment of DNA damage and oxidative stress among traffic conductors and coal miners

**DOI:** 10.12669/pjms.37.2.2848

**Published:** 2021

**Authors:** Irfan Ullah, Muhammad Zahid, Muhammad Jawad, Aatik Arsh

**Affiliations:** 1 Irfan Ullah, Kabir Medical College, Gandhara University, Peshawar, Pakistan; 2 Muhammad Zahid Department of Zoology, Islamia College, Peshawar, Pakistan; 3 Muhammad Jawad, Islamia College, Peshawar, Pakistan; 4 Aatik Arsh KMU Institute of Physical Medicine and Rehabilitation, Khyber Medical University, Peshawar, Pakistan

**Keywords:** Antioxidants, Blue-collar workers, DNA, Oxidative Stress, Pollution

## Abstract

**Objective:**

To assess the DNA damage and oxidative stress among traffic conductors and coal miners.

**Methods:**

An analytical cross-sectional survey was conducted in Karak, Pakistan from March to October 2019. A total of 240 individuals participated in the study with an age range between 17 to 55 years. Among the total sample, 60 participants had exposure to traffic pollution while 60 were mine workers. Two control groups, consisting of 60 individuals each, were also recruited for comparison with the two exposure groups. Comet assay protocols were performed for assessing DNA damage and oxidative stress (length of DNA tail, levels of Superoxide Dismutase (SOD), Malondialdehyde (MDA) and Glutathione (GSH)). Data was analyzed using T-test on statistix 9.0 software.

**Results:**

The DNA tail length in traffic conductors ranged from 26.83-30.55µm (Mean=28.69 µm while their control group had DNA tail length of 7.98-9.26µm (Mean= 8.62). There was significant difference (P <0.001) between exposure and control group. The DNA length recorded in coal mine workers and their control group was ranged from 29.06-31.26µm (Mean=30.16µm) and 9.42-10.22µm (Mean=9.82), respectively. There was significant difference (P <0.001) between the two groups. As compared to control groups, both exposure groups have high levels of Superoxide Dismutase and Malondialdehyde and low levels of Glutathione. The finding was statistically significant (P <0.001).

**Conclusion:**

Increased inhalational exposure to air pollutants via working in traffic or coal mines can impose higher oxidative stress and DNA damage among workers as compared to the general population.

## INTRODUCTION

Air pollution can pose hazardous impact on health and has the capacity to disturb life.^1^ Particulate matter and pollutants can be produced particularly in larger amounts while mining via upper surface removal of mine rocks, blasts done inside mines and loading materials on to vehicles.^2^ Similarly, large amount of pollutants are produced by vehicles.^2^,^3^

Pollutants like particulate matter 2.5 (PM 2.5) can present in the form liquid as droplets or solid of various sizes.^4^ They are defined as PM with an aerodynamic diameter of smaller than 2.5 μm.^5^ PM 2.5 contributes towards the production of high number of reactive oxygen species (ROS) including hydroxyl radicals because heavy metals can adsorb onto the pores and surface of the particles. Its smaller size makes it difficult to be expelled by the respiratory mucociliary cells whereas another pollutant PM 10, found in the air, can be cleared easily from airway when deposited in upper respiratory tract due to its larger size.^6^

The oxidative capacity of PM 2.5 can be assessed by its ability to form hydroxyl radicals (-OH) in the presence of hydrogen peroxide which in turns plays an active role in inducing oxidative stress.^7^ This increased concentration of free radicals can lead to lipid peroxidation and cause cell and DNA damage inside the human body. This DNA damage can be measured as length of DNA tail using comet assay. The longer the length of the DNA tail, the higher the damage to the DNA.^8^

Despite the fact that many international studies highlighted the fact that pollutants can cause oxidative stress and DNA damage, however there is scarce literature available regarding it in Pakistan. Therefore current study was designed to assess oxidative stress and DNA damage caused by PM 2.5 exposure among traffic conductors and coal miners of Karak, Pakistan.

## METHODS

An analytical cross-sectional survey was conducted in Karak after the Bioethical Approval (319/2019/ICP) by Islamia College, Peshawar, from March to October 2019. There were two main groups; exposure and control group. In the exposure group, those participants were included who had direct exposure to PM 2.5. Traffic conductor and coal miners who worked for at least six hours per day for six days a week were kept in exposure group. In the control group, participants from general population were recruited who were not directly exposed to air pollution. Individuals with history of smoking, drug addiction, diabetes, hypertension and any other major medical complication were excluded from the study.

A total of 240 people was included in the study with an age range between 17 to 55 years: 60 working in mines, 60 working as traffic conductors and two control groups consisting of 60 participants each for comparison to exposure groups. After obtaining written consent from each participant, a demographic questionnaire was filled and 3ml blood sample was drawn per participant in both exposure and control groups. Sample was taken in EDTAK2 tube and kept at 4°C temperature in a freezer. Samples were then transported to a molecular laboratory within 3-4 hours to perform comet assay and protocol for assessing parameters of damage to DNA and oxidative stress (length of DNA tail, levels of Superoxide Dismutase (SOD), Malondialdehyde (MDA) and Glutathione (GSH)). Demographic and lab data was analyzed using statistix 9.0 software and t-test was applied.

## RESULTS

The DNA tail length in traffic conductors ranged from 26.83-30.55µm (Mean=28.69 µm while their control group had DNA tail length of 7.98-9.26µm (Mean=8.62). There was significant difference (P <0.001) between exposure and control group. The DNA length recorded in coal mine workers and their control group was ranged from 29.06-31.26µm (Mean=30.16µm) and 9.42-10.22µm (Mean=9.82), respectively. There was significant difference (P<0.001) between the two groups.

The concentration of GSH in traffic conductors and coal miners was significantly (P<0.001) lower as compared to their respective controls. On the other hand, concentration of SOD and MDA were significantly (P <0.001) higher in traffic conductors and coal miners as compared to their respective controls (Table-I).

**Table-I:** DNA tail length, Glutathione, Superoxide Dismutase and Malondialdehyde in Control and Exposure groups.

*Outcome*	*Variable*	*Mean*	*Minimum*	*Maximum*	*P-value*
DNA tail	Control for traffic conductors	8.6283	7.9892	9.2675	< 0.01
	Traffic conductors	28.693	26.834	30.553	
	Control for coal miners	9.8247	9.4204	10.229	< 0.01
	Coal miners	30.163	29.060	31.266	
GSH	Control for traffic conductors	62.755	62.286	63.224	< 0.01
	Traffic conductors	51.827	51.100	52.554	
	Control for coal miners	62.723	62.267	63.179	< 0.01
	Coal miners	51.830	51.103	52.557	
SOD	Control for traffic conductors	57.995	57.518	58.473	< 0.01
	Traffic conductors	67.875	67.155	68.595	
	Control for coal miners	57.942	57.439	58.446	< 0.01
	Coal miners	67.918	67.184	68.652	
MDA	Control for traffic conductors	7.3625	6.9684	7.7566	< 0.01
	Traffic conductors	9.9403	9.3987	10.482	
	Control for coal miners	7.3592	6.9662	7.7521	< 0.01
	Coal miners	9.9422	9.4000	10.484	

GSH: Glutathione, SOD: Superoxide Dismutas, MDA: Malondialdehyde

## DISCUSSION

Abundant evidence on the adverse health effects associated with the exposure to particulate matter, especially PM 2.5, exists in literature. The toxic effects of PM 2.5 are mediated by its ability to attach heavy metals onto its surface.^9^-^11^ The most common examples being heavy metals like chromium and vanadium, that mediate oxidative stress and DNA damage by adsorbing onto PM 2.5 particles.^12^,^13^ The oxidation of these metals produces ROS within the cell cytoplasm and cause tissue damage by affecting cellular organelles and nuclear material.^14^,^15^ It also promotes activation of intracellular oxidant pathways that indirectly induce toxic responses.^16^-^18^

This genetotoxic effect of PM 2.5, assessed in this study using comet assay protocol, found that the means of DNA tail length of traffic contractors and coal miners were significantly larger than their respective controls. Similar, results regarding the genetotoxic role of PM 2.5 have been reported previously by Bonetta et al. and Ateeq et al. thus supporting the findings of the current study.^19^-^21^

The GSH concentration was noted to be higher in control groups of both traffic and coal workers as compared to the exposed groups. This low level of GSH in exposed individuals may be attributed to the PM2.5. The finding of lower GSH levels due to air pollution is supported by results of Ateeq et al. and Harris and Shi, who found lower GSH values in participants exposed to air pollution as compared to controls.^21^,^22^

In contrast, the SOD and MDA concentration in the current study were found to be higher in traffic contractors and coal miners as compared to the control groups. This increased level of both SOD and MDA positively correlated with the inhaled PM2.5. The elevated MDA concentrations inside the body in exposed individuals dictate the extent of oxidative stress, thus indicating higher risk of oxidative stress in the exposed group.^21^

### Limitation of the study

Despite the fact that current study was a preliminary study conducted in Pakistan, which reported devastating effects of air pollutants in traffic and coal mine workers, yet it has some limitations. Current study was conducted in one district only and had small sample size, due to which generalizability of the results of current study is questionable. Moreover, current study was only limited to traffic and coal mine workers and did not assess general population.

## CONCLUSION

It can be concluded that increased inhalational exposure to air pollutants in individuals working in traffic or coal mines can impose higher oxidative stress and DNA damage as compared to the general population. Therefore, it is essential that precautionary and protective measures must be undertaken to minimize exposure by promoting work place safety to avoid unwarranted detrimental effects on health.

### Authors’ Contributions:

**IU:** Concept and study design, literature search and literature review, drafting the manuscript, responsible and accountable for the accuracy and integrity of the work.

**MZ:** Concept and study design, critical revision, final approval of the version to be published.

**MJ:** Literature search and literature review, acquisition of data, drafting the manuscript.

**AA:** Literature search and literature review, drafting the manuscript, data analysis and interpretation.
